# Transcriptome Changes in *Eriocheir sinensis* Megalopae after Desalination Provide Insights into Osmoregulation and Stress Adaption in Larvae

**DOI:** 10.1371/journal.pone.0114187

**Published:** 2014-12-03

**Authors:** Min Hui, Yuan Liu, Chengwen Song, Yingdong Li, Guohui Shi, Zhaoxia Cui

**Affiliations:** 1 EMBL, Institute of Oceanology, Chinese Academy of Sciences, Qingdao, 266071, China; 2 University of Chinese Academy of Sciences, Beijing, 100049, China; 3 National & Local Joint Engineering Laboratory of Ecological Mariculture, Qingdao, 266071, China; Public Health Research Institute at RBHS, United States of America

## Abstract

*Eriocheir sinensis*, an extremely invasive alien crab species, has important economic value in China. It encounters different salinities during its life cycle, and at the megalopal stage it faces a turning point regarding the salinity in its environment. We applied RNA sequencing to *E. sinensis* megalopae before (MB) and after (MA) desalination, resulting in the discovery of 21,042 unigenes and 908 differentially expressed genes (DEGs, 4.32% of the unigenes). The DEGs primarily belonged to the Gene Ontology groups “Energy metabolism,” “Oxidoreductase activity,” “Translation,” “Transport,” “Metabolism,” and “Stress response.” In total, 33 DEGs related to transport processes were found, including 12 proton pump genes, three *ATP-binding cassettes* (*ABCs*), 13 *solute carrier* (*SLC*) family members, two *sweet sugar transporter* (*ST*) family members and three other substance transporters. Mitochondrial genes as well as genes involved in the tricarboxylic acid cycle, glycolytic pathway, or β-oxidation pathway, which can generate energy in the form of ATP, were typically up-regulated in MA. 11 unigenes related to amino acid metabolism and a large number of genes related to protein synthesis were differentially expressed in MB and MA, indicating that *E. sinensis* possibly adjusts its concentration of free amino acid osmolytes for hyper-osmoregulation. Additionally, 33 salinity and oxidative stress induced genes were found to be differentially expressed, such as the *LEA2*, *HSPs*, *GST* and coagulation factor genes. Notably, *LEA2* is an extremely hydrophilic protein that responds to desiccation and reported for the first time in crabs. Therefore, we suppose that when the environment is hypo-osmotic, the megalopae might compensate for ion loss via hyper-osmoregulation by consuming more energy, accompanied by a series of stress induced adaptions. This study provides the first genome-wide transcriptome analysis of *E. sinensis* megalopae for studying its osmoregulation and stress adaption mechanisms.

## Introduction

Salinity is an important environmental factor influencing aquatic organisms. Their adaption to fluctuations in salinity is a complicated process with different mechanisms; osmoregulation is one of the most significant of these processes for many animal species, including decapod crustaceans. The Chinese mitten crab *Eriocheir sinensis* (H. Milne Edwards, 1853) can serve as a model species for the study of salinity adaption and osmoregulation in decapods.

As an anadromous species, *E. sinensis* confronts a variety of different ambient salinities during its life cycle making it a typical euryhaline crab species [1], [2]. In the natural environment, *E. sinensis* juveniles migrate from the sea to fresh water and spend the majority of their life in fresh water. Therefore, in order for *E. sinensis* to accumulate megalopae for their transition to freshwater conditions during juvenile culturing, salinity reduction (desalination) is essential, thus, seawater is commonly diluted at the beginning of the megalopae stage. At this point, the megalopae can be sold to farmers for freshwater pond cultures [3], [4]. This process requires that the larval crabs adapt to salinity changes and regulate their hemolymphatic osmotic pressure via osmoregulation. Therefore, knowledge of the molecular mechanisms employed by *E. sinensis* to address different salinities is important for both understanding the adaption of *E. sinensis* to its environment and improving larvae culture conditions.

Most studies on decapod osmoregulation have focused on the organs involved [5], the patterns of osmoregulation, and body fluid composition and concentration [6], with consideration of the osmotic and ionic gradients against the external medium. The mechanisms typically refer to ion movement and the location of ion pumps [7], [8]. At the molecular level, osmoregulation-related genes and their expression have been reported in several species. For example, in the adult of *E. sinensis*, Glutamate Dehydrogenase is found to serve important functions in controlling osmoregulation [9]. In *Litopenaeus vannamei*, the expression of Na^+^/K^+^-ATPase is stimulated by salinity stress, suggesting that Na^+^/K^+^-ATPase plays an role in adjusting ion density and osmoregulation [10]. In *Portunus trituberculatus* exposed to different salinity levels, 417 DEGs were detected by a cDNA microarray chip [11]. More recently, with the advent of next-generation RNA sequencing (RNA-Seq) technology, transcriptomic analyses have been performed in decapod species in order to obtain molecular information related to osmoregulation and salinity stress, such as in *P. trituberculatus* adults [12] and even *E. sinensis* [13], and many more DEGs have been identified.

However, the transcriptomic studies on decapod salinity adaption and osmoregulation have been only performed in adults and the samples have been generally challenged with high salinity. Only limited data are available for larvae and there are notably few recent studies of larval adaption to low-salinity levels. The osmoregulatory adaption utilized by adults do not necessarily reflect those employed by larvae and osmoregulatory patterns could change after the metamorphosis from larvae to adults [14]. As planktonic organisms, larvae survive in completely different environments from benthic adults [15]. Additionally, in nature, mitten crab megalopae begin to migrate into estuaries and move further upstream to rivers, indicating that a more sophisticated adaption mechanism is formed at the megalopal stage. In the study of osmoregulation during the ontogeny of *E. sinensis* [16], [17], megalopal stage has been revealed to be crucial for the development of adult osmoregulation pattern, and a moderately hyper-/hypo-regulating mechanism in megalopae is proposed. However, molecular knowledge for this issue is inadequate.

In this study, our goal was to detail the molecular basis of osmoregulation and the stress adaption mechanisms of larvae at key developmental stages with a comparative transcriptomic analysis of *E. sinensis* megalopae before and after desalination. Considering the ecological and economic importance of the species [18], [19], molecular knowledge of their environmental tolerance and the physiological changes in their larvae can be valuable for their management and potential culture.

## Materials and Methods

### Ethics Statement

The sampling location is not privately owned or protected, and no specific permission is required. No endangered or protected species were involved in the study. The experiments were performed in strict accordance with the guidelines set by the Institutional Animal Care and Use Committee (IACUC) of the Chinese Academy of Sciences (No. 2011-2). This study was specifically approved by the Committee on the Ethics of Animal Experiments of the Institute of Oceanology at the Chinese Academy of Sciences. All efforts were made to minimize the suffering of the larvae. The ARRIVE (Animal Research: Reporting of *In Vivo* Experiments) Guidelines Checklist (Checklist S1) was included in the supporting information as required.

### Larval material

All of the specimens were obtained from a farm in Panjin, China in June 2013. The newly hatched larvae were cultured in a tank with 18 ppt water salinity until they molted into megalopae. In the six days that followed, the water was diluted chronologically (Day 1 - Day 6: 18, 15, 12, 9, 6, 2 ppt). The megalopae in the water with 18 and 2 ppt salinities were taken and immediately frozen in liquid nitrogen to generate the before (MB) and after (MA) desalination samples, respectively, and kept at −80°C for further use. Two replicate samples for MB and MA were established, respectively. Each sample included 50 individuals.

### cDNA library construction and RNA-Seq

The total RNA was extracted from the whole bodies of the larvae using the Trizol Kit (Invitrogen, USA) according to the manufacturer's instructions. Equal quantities of total RNA from two replicate samples were mixed to prepare the pooled RNA sample for RNA-Seq. The mRNA was purified from the total RNA and cut into 155 bp fragments using the TruSeq RNA Sample Prep Kit (Illumina). Double strand cDNAs were synthesized and sequencing adaptors were ligated per the Illumina manufacturer's protocol. After purification with AMPureXP beads, the ligated products were amplified to generate high quality cDNA libraries. For each stage, one cDNA library was prepared and sequenced by an Illumina HiSeq 2000 machine.

### Sequence assembly

Clean reads were obtained from the raw reads by filtering the adaptor sequences, low quality sequences (<Q20) and sequences shorter than 50 bp using Solexa QA [20]. The high quality trimmed reads were then *de novo* assembled into full length transcripts by using Trinity (http://trinityrnaseq.sourceforge.net/) with the default k-mer length of 25, following the strategy of Grabherr et al. [21]. Three independent modules in Trinity, Inchworm, Chrysalis, and Butterfly were applied sequentially to assemble the large sequence data into contigs, de Bruijin graphs and full-length transcripts. The MB and MA datasets were analyzed separately for further comparison and ultimately assembled together.

### Gene annotation and classification

Annotation of the transcripts was performed first by using the BlastX algorithm (*E*-value <1E-05) against the NCBI non-redundant (NR) database and the unigenes were obtained after clustering the top hit results. Different public databases were then selected for further functional annotation of the unigenes. Gene Ontology (GO) annotations were determined using Blast2GO to obtain a functional classification of the unigenes [22]. We used eggNOG (Evolutionary genealogy of genes: Non-supervised Orthologous Groups) to classify the potential functions of the unigenes based on known orthologous gene products [23]. KEGG (Kyoto Encyclopedia of Genes and Genomes) was used for investigating potential pathways for the genes [24]. EC (Enzyme Commission number) terms and KO (KEGG Orthology) numbers were generated by the KEGG analysis.

### Differential gene identification, enrichment and pathway analysis

Gene expression profiling was based on the number of reads mapped to the unigenes. RPKM (reads per kb of exon model per million mapped reads) values were used to estimate the expressed values and transcript levels. Briefly, the RPKM value was calculated based on the the number of reads mapping to each gene and the length of the gene [25]. A RPKM threshold value of 0.1 was set to detect the presence of a unigene. SeqMap [26] was used for reads mapping and then rSeq [27] was applied for RPKM based expression measurement. DEGs were identified by the DEseq program [28]. Genes with false discovery rates (FDR) <0.05 (-Log10 (0.05) = 1.3) and fold change values>2 were considered to be distinctly expressed. GO, eggNOG, KEGG Orthology (KO) and KEGG pathway enrichment analyses were also used to categorize the DEGs and detect the biological pathways they might be involved in. Processes, functions or components in the GO and KEGG pathway enrichment analyses with *p*-values less than 0.05 (-Log10 (0.05) = 1.3) were considered to be significantly different in MB versus MA. Based on public databases and the published literatures, the crucial DEGs related to substance transport, energy and substance metabolism, and stress response were manually checked. Additionally, partial amino acid sequences of the DEG encoding ATP synthase beta subunit (*ATPsyn-beta*) for seven other crustacean species were downloaded from NCBI and a NJ phylogeny tree was constructed based on the sequences using MEGA 4.0 [29].

### Quantitative real-time PCR (qRT-PCR) verification

To verify the expression level of the key DEGs and the accuracy of RNA-Seq, new MB and MA samples were selected for qRT-PCR. After total RNA from independent samples of MB and MA were extracted separately, the first-strand cDNA was synthesized by using M-MLV reverse transcriptase (Promega) and oligodT. Then, the cDNA was diluted 100 times by DEPC-treated water. The SYBR Green RT PCR assay was carried out in an ABI PRISM 7300 Sequence Detection System (Applied Biosystems). Eight pairs of gene-specific primers (Table 1) were used to amplify the partial cDNA sequences, respectively. Three biological replicates for each sample and three technical replicates were performed. The relative expression level was calculated using the 2^−ΔΔCt^ method. The *β-actin* gene was used to normalize the gene expression. The results were subjected to one way analysis of variance (one way ANOVA) using SPSS 16.0, and the *p-*values less than 0.05 were considered statistically significant.

**Table 1:** Gene expression analyzed by qRT-PCR and RNA-Seq.

Gene name	Unigene ID	Primer name	Primer sequences	Fold change (MA:MB)	
				RNA-Seq	qRT-PCR
***AVP***	comp51265_c0_seq1	AVP-F	GTGCTACGCTCGTCATCTCG	Inf	Inf
		AVP-R	CAGTAGAACGCACGGATTGG		
***ATP6V1A***	comp25755_c0_seq1	ATP6V1A -F	AGACTGTCAAGGACGGTAAG	Inf	126
		ATP6V1A -R	CCAGCTCTTTCGTAGAACTG		
***RAB1A-1***	comp8328_c0_seq1	RAB1A-1F	CGGAAAGTCGTGTCTGCTGT	Inf	Inf
		RAB1A-1R	CGTTCTCACAAGCATATCGGTC		
***SESB***	comp12445_c0_seq1	SESB-F	GTTACCACGCCAGAACGAGC	Inf	153
		SESB-R	CCACCTCGGACAACAGCAAC		
***GLYS***	comp45386_c0_seq1	GLYS -F	GCTGTCACCAAGTCTTTACG	0.365	0.400
		GLYS -R	GGTCCACAGCATCGTCACAC		
***GLDC***	comp38932_c0_seq2	GLDC -F	CTCAAGAGCATCGCCAATCG	0.271	0.188
		GLDC -R	TCCACAGTCTCGTCCAATGC		
***LEA2***	comp29147_c0_seq1	LEA2-F	AGCGTCTAGCAGGATTGGTC	0	0
		LEA2-R	GATTCGGAAGGTCCCACGAC		
***PCE***	comp35865_c0_seq1	PCE-F	GGTTGCCAGAGCCCTCATAC	8.863	6.122
		PCE-R	CTCGTGCCGTGCTGACAGAA		

‘Inf’ means that the genes were only expressed in MA.

## Results and Discussion

The transcriptomic sequences of *E. sinensis* megalopae before and after desalination were obtained using the Illumina sequencing platform. By comparative transcriptomic analysis, sets of genes and pathways that responded to salinity changes, especially low osmolality, were identified for the first time in crab larvae; the information acquired in this study also led to a helpful understanding of the complex stress response of *E. sinensis*.

### Transcriptome sequencing, assembly, gene annotation and classification

To identify the genes related to the adaption of *E. sinensis* larvae to low salinity, the transcriptomes were sequenced separately for megalopae before (MB: 18 ppt salinity) and after desalination (MA: 2 ppt salinity). In total, 63,433,542 and 61,764,914 raw reads were obtained from the MB and MA transcriptomes with GC percentages of 51.64% and 49.69%, respectively (Table S1). All of the raw reads were deposited into the Sequence Read Archive (SRA) database (http://www.ncbi.nlm.nih.gov/Traces/sra/) under accession numbers SRX495634 (MB) and SRX501787 (MA). The Q20 percentages were 97.35% and 97.41% for the MB and MA raw data, respectively. After trimming, 58,339,580 and 56,923,344 clean reads remained with 5.70 Gb and 5.56 Gb of data for MB and MA, respectively (Table S1). After combining MB and MA, 243,345 contigs were generated, with an average length of 367 bp and an N50 of 648 bp. After assembly, 127,983 transcripts were obtained with an average length of 1,018 bp and an N50 of 2,314 (Table S1). These data greatly enriched the genetic resources for *E. sinensis*, especially for megalopae, which can facilitate further molecular studies on mitten crabs at different developmental stages.

After the BlastX searches against the NR database, the transcript sequences were further clustered according to the top hits found, and 21,042 unigenes were obtained with annotations (*E*-value <1E-05). Compared with a previous comparative transcriptomic study in *E. sinensis* [13], a greater number of unigenes were obtained in our study, which might be because the whole bodies of larvae were used and more tissues were included. The range of our unigene lengths was from 201 bp to 22,554 bp (Figure S1) with an average length of 1,635 bp and an N50 of 2,629 bp (Table S1).

To classify the genes, all of the 21,042 annotated unigenes were analyzed for their associated GO terms, KEGG terms and eggNOG terms (Table S1). In the GO analysis, 7,499 (35.64%) of the unigenes were annotated and divided into three categories: ‘Biological process’, ‘Cellular component’ and ‘Molecular function’ (Figure S2). The number of GO terms per unigene varied from 1 to 72, and 5,300 (70.68%) of the matched unigenes were assigned to more than one GO term. In the KEGG analysis, 8,653 (41.12%) unigenes had KO numbers, and among these, 3,508 (40.54%) of the matched unigenes were assigned corresponding EC numbers. In the eggNOG analysis, 20,139 unigenes (95.71%) were properly assigned and grouped into 25 functional categories (Figure S3). Excluding genes annotated as ‘Function unknown’ and ‘General function prediction only’, the largest proportion of assignments was ‘Signal transduction mechanisms’ with 3,312 (13.67%) unigenes, followed by ‘Posttranslational modification, protein turnover, chaperones’ with 1,864 (7.70%) unigenes, and ‘Transcription’ with 1815 (7.50%) unigenes (Figure S3). Genes related to substance transport were generally included in the ‘Carbohydrate transport and metabolism’, ‘Inorganic ion transport and metabolism’, ‘Amino acid transport and metabolism’, and ‘Lipid transport and metabolism’ groups, accounting for 3.90%, 2.82%, 2.71% and 2.58% of the unigenes, respectively (Figure S3). These annotations and classifications supplied an overall framework for the megalopal transcriptome as well as background for the following DEG analysis.

A primary objective for the transcriptomic sequencing was to identify the genes involved in osmoregulation. In the transcriptomes, many of the genes involved in ion transport processes were identified, such as genes encoding the Na^+^/Ca2^+^ exchanger, Na^+^/K^+^ ATPase, Na^+^/K^+^/2Cl^−^ cotransporter, Na^+^/dicarboxylate, Na^+^/tricarboxylate and phosphate transporter, V-type proton ATPase, solute carrier family members, and the organic cation transporter, all of which potentially play roles in osmoregulation.

### Differentially expressed gene enrichment and preference

**Figure 1:**
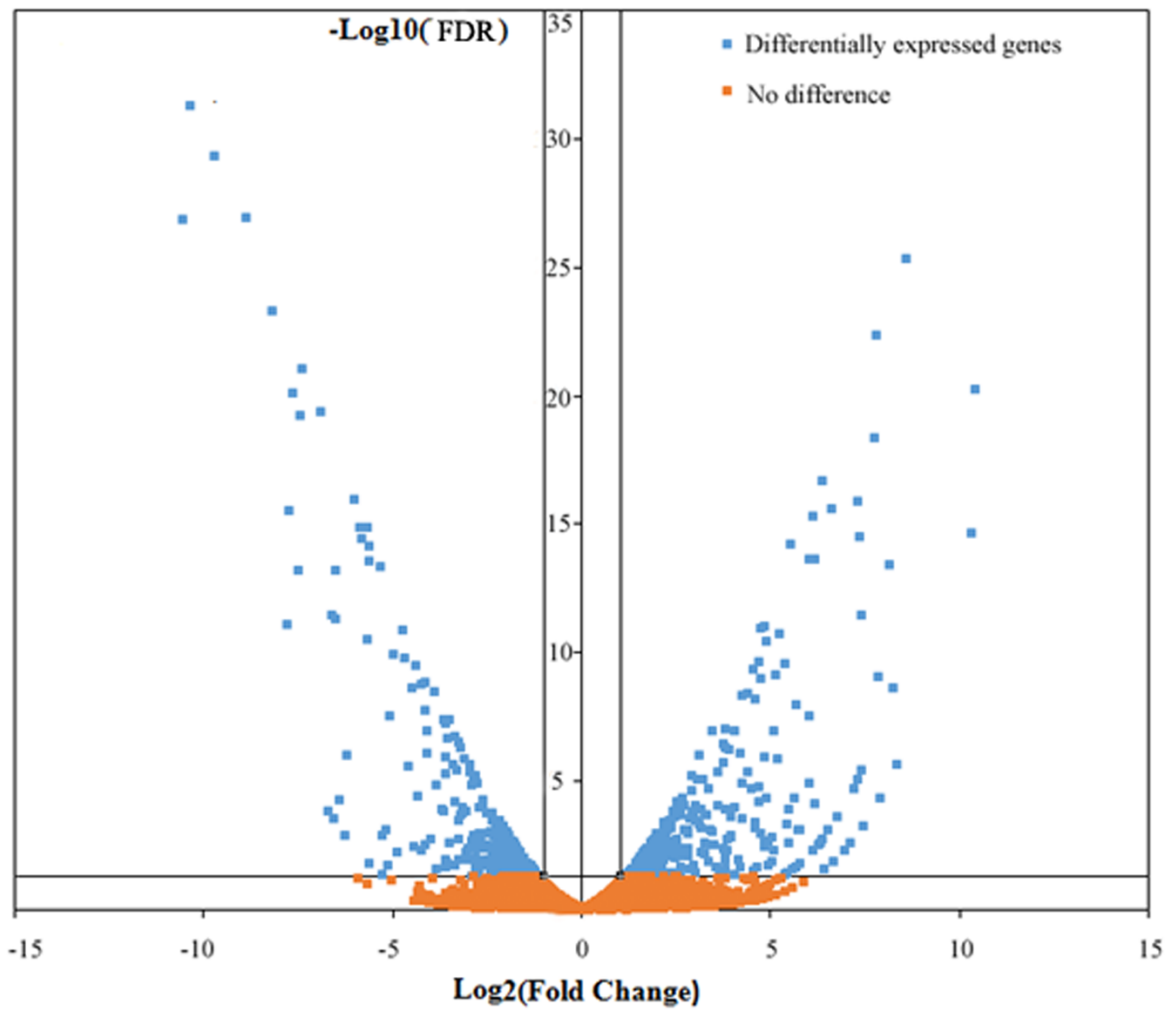
Volcano plot of differentially expressed genes (DEGs) from the transcriptomes of MB and MA in *Eriocheir sinensis*. For each unigene, the ratio of expression levels (MB vs. MA) was plotted against the -log error rate. The horizontal line indicates the significance threshold (FDR <0.05), and the vertical lines indicate the two fold change threshold. Non-differentially expressed genes are shown with orange dots, and DEGs are shown with blue dots.

Overall, after water desalination (18 ppt to 2 ppt), the expression of 908 of the genes (4.32% of all unigenes) in MA changed significantly compared with MB (Table S2), including 538 significantly up-regulated and 370 down-regulated genes, which indicates that changes in a relatively small percentage of the genes are responsible for adaption. A distribution of the significant changes in expression is illustrated in a volcano plot (Figure 1). Notably, 193 and 16 unigenes were specifically expressed in MA and MB (Table S2), respectively, which might play important roles in adaption to changes in salinity.

**Figure 2:**
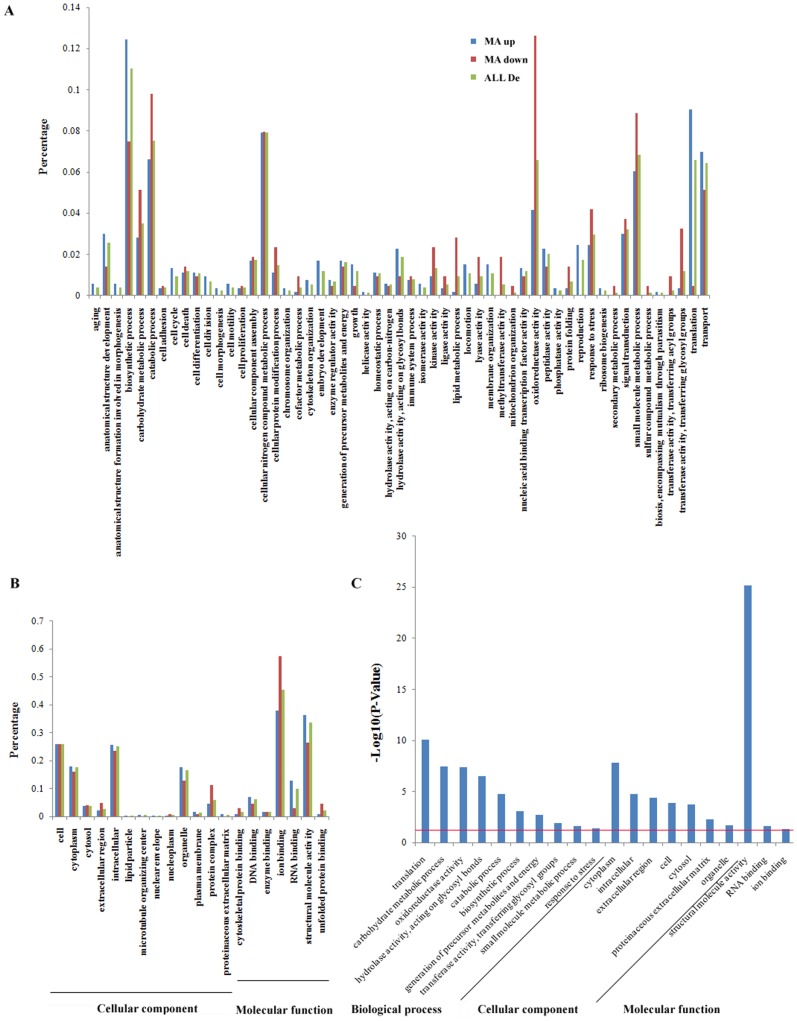
GO distributions of the differentially expressed genes (DEGs) from the transcriptomes of MB and MA in *Eriocheir sinensis*. (A) Different functional distribution of the DEGs involved with biological processes; (B) Different functional distribution of the DEGs involved with cellular components and molecular functions; (C) Differentially expressed functional processes. The horizontal line indicates the significance thresholds (*p*<0.05).

The functional distribution of the DEGs in various processes was analyzed by GO, KEGG and eggNOG enrichment. In the GO enrichment, 512 DEGs with GO terms were categorized into different functional groups. In the ‘Biological process’ group, the first 10 processes were ‘Biosynthetic process (11.02%)’, ‘Cellular nitrogen compound metabolic process (7.93%)’, ‘Catabolic process (7.52%)’, ‘Small molecule metabolic process (6.85%)’, ‘Oxidoreductase activity (6.59%)’, ‘Carbohydrate metabolic process (3.49%)’, ‘Translation (6.59%)’, ‘Transport (6.45%)’, ‘Signal transduction (3.22%)’, and ‘Response to stress (2.96%)’ (Figure 2A). In the ‘Cell component’ group, the main components were ‘Cell (25.81%)’ and ‘Intracellular (25.16%)’ (Figure 2B), while in the ‘Molecular function’ group, ‘Ion binding (45.41%)’ covered the largest number of DEGs followed by ‘Structural molecule activity (33.67%)’ (Figure 2B). Many of these GO terms are also common in the transcriptomic study for *E. sinensis* adult treated with high salinity [13]. After the overall comparison was completed, the top 20 significantly changed categories were obtained (*p*<0.05; Figure 2C), including ‘Hydrolase activity, acting on glycosyl bonds’ and ‘Ion binding’, which might be induced by changes in salinity.

**Figure 3:**
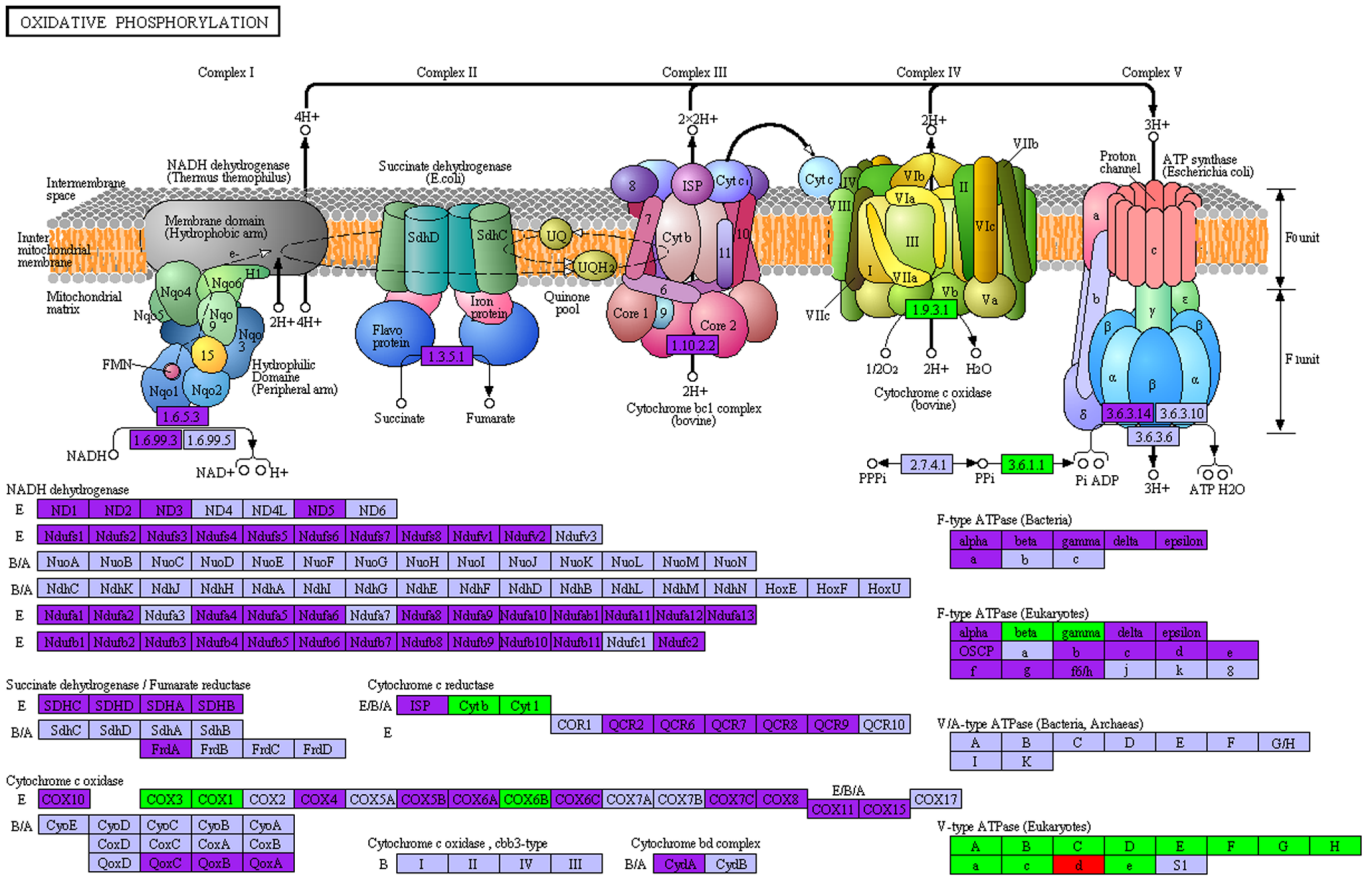
Oxidative phosphorylation pathway. The pathway is based on a KEGG pathway analysis. The up-regulated and down-regulated genes are labeled by green and red, respectively, and the purple color represents genes with no expression differences between the MB and MA transcriptomes of *Eriocheir sinensis*.

In the KEGG enrichment, 333 DEGs with KO terms were assigned to six groups including 38 potential categories (Figure S4). The categories with the most DEGs when comparing MB with MA were ‘Infectious Diseases (11.18%)’, ‘Translation (10.92)’, ‘Transport and Catabolism (5.72%)’, ‘Lipid Metabolism (5.33%)’, ‘Amino Acid Metabolism (5.19%)’ and ‘Energy Metabolism (5.06)’ (Figure S4). The significantly distinct categories (*p*<0.05) were ‘Carbohydrate Metabolism’, ‘Energy Metabolism’, ‘Lipid Metabolism’, ‘Xenobiotics Biodegradation and Metabolism’, ‘Metabolism of Terpenoids and Polyketides’, ‘Translation’, ‘Transport and Catabolism’, ‘Excretory System’ and ‘Environmental Adaption’, ‘Immune Diseases’ and ‘Infectious Diseases’ (Figure S5). The DEGs were predicted in the following specific pathways: 97 DEGs were involved in ‘Metabolic pathways’, 75 in ‘Ribosome’, 27 in ‘Biosynthesis of secondary metabolites’, 20 in ‘Oxidative phosphorylation’ and many others in pathways related to amino acid and fatty acid metabolism. One crucial pathway, ‘Oxidative phosphorylation’, was selected for further study, which was also found to be one of the top pathways for high salinity change of *E. sinensis* adult [13]. All of the genes with KO terms in MA and MB were subjected to a KEGG pathway analysis combining up- and down-regulated genes; these are labeled with different colors in Figure 3 (Purple: no expression difference; Green: up-regulated in MA; Red: down-regulated in MA). With respect to ‘Oxidative phosphorylation’, the majority of the genes located at 105 positions during this process were matched and most of the subunits of V-type ATPase were found to be up-regulated. The other DEGs largely encode Cytochrome c reductase or oxidase, whose activity has been reported to be influenced by environmental osmolality in the *E. sinensis* adult [30].

In the eggNOG analysis, excluding ‘Function unknown (23.42%)’ or ‘General function prediction only (7.43%)’ genes, 820 DEGs were principally involved in the processes of ‘Translation, ribosomal structure and biogenesis (9.37%)’, ‘Signal transduction mechanisms (9.16%)’, ‘Carbohydrate transport and metabolism (7.94%)’, ‘Energy production and conversion (4.89%)’, and ‘Amino acid transport and metabolism(4.28%)’ (Figure S6).

When the three different enrichment methods are combined, the common enrichment categories for the DEGs are ‘Energy metabolism’, ‘Oxidoreductase activity’, ‘Translation’, ‘Transport’, ‘Amino acid metabolism’, ‘Carbohydrate metabolism’, ‘Lipid metabolism’ and ‘Stress response’, indicating that the involved genes are generally related to energy and substance metabolism and transport, as well as stress stimulation. The above enrichment methods facilitated an investigation of gene function in the following analysis.

### DEGs associated with osmoregulation and stress adaption

According to the classification above and by manual inspection, 105 key unigenes possibly related to osmoregulation or stress response were identified from the 908 DEGs and divided into the following three categories (summarized in Tables 2, 3, and 4).

**Table 2:** Differentially expressed genes related to substance transport in the megalopae of *Eriocheir sinensis* before (MB) and after (MA) desalination.

Gene name	Unigene ID	Protein name	Matched Organism	Fold change (MA:MB)*
**Proton pump**	**12**			
*AVP*	comp51265_c0_seq1	pyrophosphate-energized proton pump-like	*Xenopus (Silurana) tropicalis*	**Inf**
*ATP6V1D1*	comp43014_c1_seq1	Vacuolar proton pump subunit D1	*Harpegnathos saltator*	**2.933**
*ATP6V1A*	comp43434_c0_seq1	V-type proton ATPase catalytic subunit A	*Crassostrea gigas*	**2.279**
*ATP6V1E*	comp46348_c1_seq1	V-type proton ATPase subunit E	*Danaus plexippus*	**2.984**
*ATP6V1H-1*	comp36797_c0_seq	V-type proton ATPase subunit H isoform 1	*Nasonia vitripennis*	**3.134**
*ATP6V1A*	comp25755_c0_seq1	V-type proton ATPase catalytic subunit A-like	*Megachile rotundata*	**Inf**
*ATP6V0D*	comp44843_c3_seq1	V-ATPase subunit D	*Locusta migratoria*	0.087
*ATP6V0A*	comp44902_c2_seq3	V-H-ATPase subunit A	*Litopenaeus vannamei*	**3.330**
*ATP6V1G*	comp41018_c2_seq5	vacuolar ATP synthase subunit G-like	*Acyrthosiphon pisum*	**2.800**
*VATB*	comp40488_c0_seq1	vacuolar ATP synthase subunit B K form	*Carcinus maenas*	**3.278**
*ATPsyn-beta*	comp15007_c0_seq1	ATP synthase beta subunit	*Maconellicoccus hirsutus*	**Inf**
*ATP13A1*	comp32933_c0_seq3	ATPase type 13A1	*Helicoverpa zea*	**2.504**
***ABC*** ** family**	**3**			
*ABC*	comp45288_c0_seq3	ABC protein, subfamily ABCG	*Daphnia pulex*	**5.547**
*ABC*	comp45124_c0_seq2	ABC protein, subfamily ABCG	*Daphnia pulex*	0.353
*ABCD-3*	comp47981_c0_seq1	ATP-binding cassette sub-family D member 3	*Crassostrea gigas*	0.309
***SLC*** ** family**	**12**			
*SLC2A1*	comp44861_c1_seq1	solute carrier family 2, facilitated glucose transporter member 1-like	*Apis florea*	0.321
*SLC6A5*	comp42751_c0_seq1	solute carrier family 6, member 5	*Branchiostoma floridae*	0.213
*SLC10A2*	comp30614_c0_seq1	ileal sodium/bile acid cotransporter-like	*Xenopus (Silurana) tropicalis*	**2.347**
*SLC16A (MCT)*	comp45040_c0_seq1	monocarboxylate transporter	*Aedes aegypti*	**4.045**
*SLC17A5*	comp45920_c4_seq2	sialin-like	*Nasonia vitripennis*	**2.911**
*SLC17A5*	comp48188_c0_seq3	sialin, putative	*Ixodes scapularis*	0.228
*SLC22A (ORCT)*	comp37144_c0_seq1	organic cation transporter, putative	*Pediculus humanus corporis*	**8.484**
*SLC22A (ORCT)*	comp47182_c1_seq1	organic cation transporter protein-like	*Bombus impatiens*	0.393
*SLC25A10 DIC*	comp45969_c3_seq2	mitochondrial dicarboxylate carrier, putative	*Pediculus humanus corporis*	0.147
*SLC26A11*	comp37309_c0_seq1	sodium-independent sulfate anion transporter-like	*Acyrthosiphon pisum*	0.236
*SLC34A2*	comp20651_c1_seq1	sodium-dependent phosphate transport protein 2B-like	*Ciona intestinalis*	**Inf**
*SLC46A3*	comp44011_c0_seq1	solute carrier family 46 member 3-like	*Acyrthosiphon pisum*	0.256
*SLC18B1-CF192*	comp12124_c0_seq1	MFS-type transporter C6orf192-like	*Acyrthosiphon pisum*	0.378
***Sweet sugar transporter*** ** family**	**2**			
*ST*	comp47826_c0_seq1	sugar transporter	*Aedes aegypti*	**5.015**
	comp40265_c0_seq2	sugar transporter	*Culex quinquefasciatus*	**3.173**
**Other transporter**	**3**			
*RAB1A-1*	comp8328_c0_seq1	ras-related protein Rab-1A-like isoform 1	*Oreochromis niloticus*	**Inf**
*APOD*	comp37629_c0_seq2	apolipoprotein D-like	*Acyrthosiphon pisum*	**Inf**
*SORL*	comp44488_c0_seq3	sortilin-related receptor, L(DLR class) A repeats-containing-like apolipoprotein E	*Saccoglossus kowalevskii*	0.332

* The numbers in bold indicate gene expression was up-regulated after (MA) desalination; ‘Inf’ means that the genes were only expressed in MA.

**Table 3:** Differentially expressed genes associated with energy and substance metabolism in the megalopae of *Eriocheir sinensis* before (MB) and after (MA) desalination.

Gene name	Unigene ID	Protein name	Matched Organism	Fold change (MA:MB)*
**Mitochondrion gene**	**8**			
*COX3*	comp23951_c0_seq1	cytochrome c oxidase subunit III	*Eriocheir hupensis*	**2.661**
*COX6B1*	comp11463_c0_seq1	cytochrome c oxidase subunit VIb polypeptide 1 (ubiquitous)	*Xenopus laevis*	**Inf**
*COX1*	comp32843_c0_seq1	cytochrome c oxidase subunit I	*Eriocheir sinensis*	**2.345**
*CYTC*	comp14887_c0_seq1	cytochrome c-like	*Metaseiulus occidentalis*	**Inf**
*CYTB*	comp37963_c1_seq1	cytochrome b	*Eriocheir japonica*	**2.322**
*CYP302A1*	comp46909_c0_seq2	cytochrome P450 302a1, mitochondrial	*Nasonia vitripennis*	**5.179**
*MDH2*	comp22942_c0_seq1	malate dehydrogenase 2, NAD (mitochondrial)	*Xenopus laevis*	**Inf**
*SESB*	comp12445_c0_seq1	stress-sensitive B-like	*Saccoglossus kowalevskii*	**Inf**
**Glyco- and fatty acids- metabolism**	**18**			
*GLS*	comp45379_c0_seq1	alpha glucosidase	*Litopenaeus vannamei*	**3.644**
*NSE*	comp14951_c0_seq1	neuron-specific enolase	*Gekko japonicus*	**Inf**
*ENO*	comp14951_c1_seq1	enolase	*Clonorchis sinensis*	**Inf**
*FBA*	comp13661_c0_seq1	fructose-bisphosphate aldolase	*Lepeophtheirus salmonis*	**Inf**
*GAPDH*	comp17806_c0_seq1	glyceraldehyde-3-phosphate dehydrogenase	*Gobiocypris rarus*	**Inf**
*TPI*	comp52076_c0_seq1	triosephosphate isomerase	*Blattella germanica*	**Inf**
*GLYS*	comp45386_c0_seq1	glycogen synthase	*Danaus plexippus*	0.365
*ACADM*	comp53262_c0_seq1	medium-chain specific acyl-CoA dehydrogenase, mitochondrial	*Otolemur garnettii*	**Inf**
*ACAD9*	comp59541_c0_seq1	acyl-CoA dehydrogenase family member 9, mitochondrial	*Gallus gallus*	**Inf**
*ACADM*	comp53262_c0_seq1	medium-chain specific acyl-CoA dehydrogenase, mitochondrial	*Otolemur garnettii*	**Inf**
*ACAD-1*	comp48322_c0_seq1	similar to acyl-coa dehydrogenase isoform 1	*Tribolium castaneum*	**3.052**
*ACADL*	comp44259_c0_seq1	long-chain acyl-CoA dehydrogenase	*Xenopus (Silurana) tropicalis*	**2.357**
*ACOT3*	comp47489_c1_seq7	acyl-CoA thioesterase 3	*Rattus norvegicus*	**5.059**
*AACS*	comp40863_c0_seq1	acetoacetyl-CoA synthetase	*Columba livia*	0.210
*ACD9*	comp42655_c4_seq1	acyl-CoA delta-9 desaturase	*Eriocheir sinensis*	0.097
*FCS*	comp49033_c0_seq1	fatty acid synthase	*Litopenaeus vannamei*	0.217
*sPLA2*	comp44696_c0_seq1	similar to secretory Phospholipase A2, partial	*Tribolium castaneum*	0.246
*PLA2*	comp46303_c3_seq2	phospholipase-like protein A2, group	*Daphnia pulex*	0.227
**Amino acids metabolism & protein synthesis**	**13**			
*PRODH1*	comp30592_c0_seq1	proline dehydrogenase 1, mitochondrial-like isoform X2	*Ceratitis capitata*	**Inf**
*GATM*	comp21253_c1_seq1	glycine amidinotransferase, mitochondrial-like	*Ciona intestinalis*	**Inf**
*GLUS*	comp49678_c0_seq1	glutamine synthetase	*Aiptasia pallida*	**Inf**
*PRKC*	comp50314_c0_seq1	protein kinase C-like 2-like	Hydra magnipapillata	**Inf**
*NACA*	comp11587_c0_seq1	nascent polypeptide-associated complex subunit alpha, putative proteostasis	Pediculus humanus corporis	**Inf**
*FOLH1*	comp43708_c0_seq1	glutamate carboxypeptidase 2, partial	*Columba livia*	**2.461**
*HPD*	comp35024_c0_seq1	4-hydroxyphenylpyruvate dioxygenase	*Aedes aegypti*	0.221
*HGD*	comp43351_c1_seq1	homogentisate 1,2-dioxygenase, putative	*Pediculus humanus corporis*	0.338
*THNSL2*	comp47019_c0_seq1	threonine synthase-like 2	*Crassostrea gigas*	0.327
*HUTH*	comp40732_c0_seq1	histidine ammonia-lyase-like	*Metaseiulus occidentalis*	0.381
*GLDC*	comp38932_c0_seq2	glycine dehydrogenase	*Xenopus laevis*	0.271
*PRODH*	comp47536_c0_seq5	proline oxidase, putative	*Pediculus humanus corporis*	0.317
*GLS*	comp37741_c0_seq4	glutaminase kidney isoform, mitochondrial-like	*Nasonia vitripennis*	0.230

* The numbers in bold indicate gene expression was up-regulated after (MA) desalination; ‘Inf’ means that the genes were only expressed in MA.

**Table 4:** Differentially expressed genes associated with stress adaption in the megalopae of *Eriocheir sinensis* before (MB) and after (MA) desalination.

Gene name	Unigene ID	Protein name	Matched Organism	Fold change (MA:MB)*
**Salinity stress**	**19**			
*LEA2*	comp29147_c0_seq1	late embryogenesis abundant-like protein 2	*Brachionus plicatilis*	0
*ANXA7*	comp51373_c0_seq1	annexin A7-like	*Strongylocentrotus purpuratus*	**Inf**
*CRHBP*	comp40595_c0_seq1	corticotropin releasing hormone binding protein	*Tribolium castaneum*	**15.025**
*HSP70*	comp38390_c0_seq1	78 kDa glucose-regulated protein	*Crassostrea gigas*	**156.220**
	comp42545_c0_seq1	hsp70-1	*Ditylenchus destructor*	**167.216**
	comp17073_c0_seq1	heat shock protein 70, partial	*Dirofilaria immitis*	**Inf**
	comp32974_c0_seq3	heat shock protein 70	*Portunus trituberculatus*	0.225
*HSP90*	comp43800_c0_seq2	heat shock protein 90	*Crassostrea hongkongensis*	**167.540**
	comp45568_c2_seq4	heat shock protein 90-2	*Portunus trituberculatus*	0.391
	comp12491_c0_seq1	hsp90 protein	*Porcellio laevis*	0.173
*HSC71*	comp27906_c0_seq1	heat shock cognate 71 kDa protein	*Saimiri boliviensis boliviensis*	0.207
*HS6B*	comp35570_c0_seq1	heat shock protein 67B2	*Caligus rogercresseyi*	**4.468**
*dnaJ homolog*	comp14806_c0_seq1	dnaJ homolog subfamily C member 7-like	*Oreochromis niloticus*	**Inf**
	comp17818_c0_seq1	dnaJ homolog subfamily B member 6-like	*Nomascus leucogenys*	**Inf**
*F11*	comp46969_c5_seq6	coagulation factor XI	*Acromyrmex echinatior*	**7.526**
	comp30467_c0_seq1	coagulation factor XI	*Loxodonta africana*	**Inf**
*SRAP*	comp38828_c3_seq2	serine-rich adhesin for platelets-like	*Ceratitis capitata*	**14.540**
*CLOP*	comp45256_c0_seq1	clotting protein precursor	*Pacifastacus leniusculus*	**2.304**
*PCE*	comp35865_c0_seq1	proclotting enzyme	*Acromyrmex echinatior*	**8.863**
**Oxidative stress**	**14**			
*GST*	comp50723_c0_seq1	glutathione S-transferase isoform	*Haliotis diversicolor*	**Inf**
	comp45196_c0_seq2	glutathione S-transferase theta-1-like	*Taeniopygia guttata*	**3.123**
*Ferritin*	comp37978_c0_seq1	ferritin 3	*Eriocheir sinensis*	**3.488**
	comp38695_c0_seq1	ferritin2	*Eriocheir sinensis*	**2.859**
	comp38695_c1_seq1	ferritin1	*Eriocheir sinensis*	**2.730**
*DHDH*	comp40992_c0_seq1	trans-1,2-dihydrobenzene-1,2-diol dehydrogenase-like isoform 2	*Apis mellifera*	**2.838**
*MTP*	comp44928_c0_seq1	metalloprotease	*Riptortus pedestris*	**3.715**
*PLOD2*	comp19186_c0_seq1	procollagen-lysine,2-oxoglutarate 5-dioxygenase 2	*Rattus norvegicus*	**138.107**
*PXD*	comp20634_c0_seq1	chorion peroxidase-like	*Nasonia vitripennis*	0.088
*GPX*	comp30876_c0_seq1	glutathione peroxidase, partial	*Reishia clavigera*	0.304
*PIPOX*	comp48300_c0_seq1	peroxisomal sarcosine oxidase-like	*Maylandia zebra*	0.362
*MCO*	comp44967_c4_seq1	multicopper oxidase	*Culex quinquefasciatus*	0.277
*SELH*	comp41994_c0_seq3	selenoprotein H	*Odobenus rosmarus divergens*	0.279
*ALDH1L1*	comp41375_c0_seq1	aldehyde dehydrogenase 1 family, member L1-like	*Saccoglossus kowalevskii*	0.384

* The numbers in bold indicate that gene expression was up-regulated after (MA) desalination; ‘Inf’ means that the genes were only expressed in MA.

#### Transporters

The transporters are important osmoregulation factors, in charge of the uptake and efflux of important substances such as inorganic ions, sugars and amino acids. In total, 33 DEGs related to transport processes were found in MB and MA including 12 proton pump genes, three *ATP-binding cassettes* (*ABCs*), 13 *solute carrier* (*SLC*) family members, two *sweet sugar transporter* (*ST*) family members and three other transporters (Table 2). Among these, 21 genes were up-regulated and 12 genes were down-regulated after desalination in MA.

**Figure 4:**
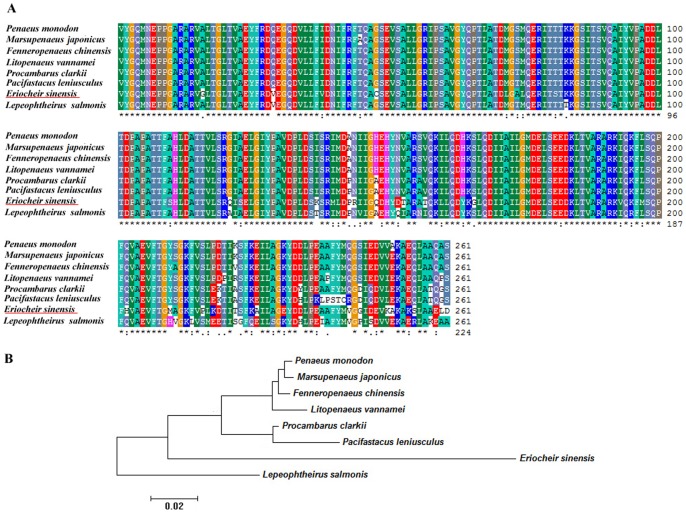
Amino acid sequence alignment of the *ATP synthase beta subunit* (A) and a phylogenetic NJ tree of the eight crustacean species based on these sequences (B).

Numerous studies have revealed that the basally located Na^+^/K^+^-ATPase drives active Na^+^ uptake from dilute media and the apically located V-ATPase complements Na^+^/K^+^-ATPase in energizing the uptake of osmoregulatory ions from highly diluted media in many euryhaline crabs [2]. In this study, genes encoding the different subunits of the V-type proton ATPase (except subunit D) and other proton pumps were found to be highly expressed after desalination in MA (Table 2), but no expression differences were observed in *Na^+^/K^+^-ATPase*. This demonstrates that the driving force of ion uptake in the larvae was primarily due to V-ATPase generating an H^+^ ion gradient to facilitate ion flow. Various ATPases have also been identified to respond to the fluctuation of salinities in other decapods [12], [31]. The partial amino acid sequences of the *ATP synthase beta subunit* available for eight crustacean species were aligned with high similarity (Figure 4A) and the resulting phylogenetic tree shows that with *Lepeophtheirus salmonis* (Caligidae) as an outgroup, all of the Penaeidae species cluster together; *Procambarus clarkia* (Cambaridae) and *Pacifastacus leniusculus* (Astacidae) cluster into one clade and then cluster with *E. sinensis* (Figure 4B), consistent with their taxonomic classifications and partially revealing the evolution of osmoregulation factors in these crustacean species.

In addition to ion pumps (ATPases), other important primary-active ATP-dependent transporters are members of the ABC transporter family, which include transmembrane and nucleotide-binding domains and catalyze the transport of diverse compounds [32]. In the white shrimp *L. vannamei*, ABC transmembrane transporters play an important role in the physiological changes related to metabolism and cell detoxification when they are exposed to stress [33]. Changes in the expression of three *ABC* genes (Table 2) were also identified after desalination in our study, with one up-regulated gene and two down-regulated genes, indicating that they might also be involved in osmoregulation of the *E. sinensis* larvae.

In addition to the primary-active transporters, many other transporter genes were detected to be differentially expressed in MB and MA. In the *SLC* family, five of these transporter genes (e.g., *SLC34A2*, encoding the sodium-dependent phosphate transport protein 2B) were up-regulated and eight (e.g., *SLC26A11*, encoding the sodium-independent sulfate anion transporter) were down-regulated. Most of the remaining transporter genes were highly expressed in MA, including the genes encoding sugar transporters (*ST*), the ras-related protein (*RAB1A-1*), and apolipoprotein D (*APOD*), while the sortilin-related receptor (*SORL*) was down-regulated. These genes are generally responsible for glucose, cholesterol, organic cation, sodium/anion, and sodium/bile transport. Many transporter proteins also show transcriptional upregulation upon dilution of environmental salinity in gill of green crab *C. maenas* [34]. It is therefore deduced that when the environmental osmolality is lower than that of the hemolymph, it is hypo-osmotic and crustaceans might compensate for ion loss by transport from their hemolymph, the so-called ‘compensatory process’ [35]. The differential expression of these key transporter genes potentially supports a model of decapod crustacean hyper-osmoregulation [2], [36].

#### Energy and substance metabolism

When animals adapt to low salinity and a hypo-osmotic environment, more energy is required [37], which can be produced by mitochondria and glycometabolism or fatty acid metabolism as well as amino acid metabolism. Moreover, as a result of metabolism, animals can also balance their osmolality by adjusting their concentrations of intra cellular osmolytes such as free amino acids, as was found to be the case in other crustacean species [13], [38], [39].

Many mitochondrial genes were up-regulated in MA (Table 3), including *cytochrome b* (*CYTB*), *cytochrome c* (*CYTC*), *cytochrome P450* (*CYP302A1*), *malate dehydrogenase 2* (*MDH2*), and *stress-sensitive B* (*SESB*). Many mitochondrial genes have also been found to express differently when confront with salinity change in *E. sinensis* adult [13]. Cytochromes b and c and P450 are all components of the electron transport chain in the inner mitochondrial membrane, which serves as a site of oxidative phosphorylation (Figure 3) and creates the electrochemical proton gradient driving ATP synthesis [40], while stress-sensitive B catalyzes the exchange of ADP and ATP across the mitochondrial inner membrane reported in *Drosophila* [41]. Moreover, MDH2 can catalyze the reversible oxidation of malate to oxaloacetate, utilizing the NAD/NADH cofactor system in the Tricarboxylic Acid Cycle (TCA) [42], which plays a central role in the catabolism of organic fuel molecules such as glucose and other sugars, fatty acids, and some amino acids. Both oxidative phosphorylation and TCA are crucial processes generating energy in the mitochondria, indicating that a large amount of energy might be necessary for low salinity adaption.

Of the DEGs related to glycometabolism and fatty acid metabolism, 18 genes were differentially expressed, with 12 up-regulated genes and six down-regulated genes in MA (Table 3). The up-regulated alpha glucosidase hydrolyzes terminal non-reducing 1-4 linked alpha-glucose residues to release a single alpha-glucose molecule [43]. Neuron-specific enolase (NSE), enolase (ENO), fructose-bisphosphate aldolase (FBA), glyceraldehyde-3-phosphate dehydrogenase (GAPDH) and triosephosphate isomerase (TPI) are all important factors involved in the glycolytic pathway generating energy in the form of ATP via oxidative splitting of glucose, which all only occur in MA, while *glycogen synthase* (*GLYS*), which is involved in the synthesis of glycogen storing energy, was down regulated. Of the genes related to fatty acid metabolism, the *ACAD* genes encoding acyl-CoA dehydrogenases were the key up-regulated genes, especially *medium-chain specific acyl-CoA dehydrogenases* (*ACADM*) and *acyl-CoA dehydrogenase family member 9* (*ACAD9*), which were found only in MA, while *Acetoacetyl-CoA synthetase* (*AACS*) was down-regulated with a fold change of 0.210 (Table 3). Acyl-CoA dehydrogenases are a class of enzymes that catalyze the initial step in each cycle of fatty acid β-oxidation. When compared with carbohydrates and proteins, fatty acids yield the most ATP via the β-oxidation pathway [44]. In contrast, *acetoacetyl-CoA synthetase* (*AACS*), *acyl-CoA delta-9 desaturase* (*ACD9*) and *fatty acid synthase* (*FCS*), which function in the biosynthesis of fatty acids, were down-regulated. In the study for the euryhaline crab *Chasmagnathus granulate*, lipids are verified to be involved in the osmotic adaption process as an energy source [45]. This reveals that the mitten crabs might also regulate the flux of multiple fuels to support the adaption process resulting from physiological variation.

With respect to amino acid metabolism, 11 unigenes were detected to be differentially expressed in MB and MA (Table 3), with four up-regulated genes and seven down-regulated genes in MA. These genes are primarily related to Glutamate, Glutamine, Glycine, Histidine, Threonine and Proline metabolism. The *glycine amidinotransferase* (*GATM*) gene plays a vital role in the creatine biosynthetic process, which is related to energy metabolism in muscle tissues [46], while the *glutamine synthetase* (*GLUS*) gene plays an essential role in the metabolism of nitrogen by catalyzing the condensation of glutamate and ammonia to form glutamine [47]. Notably, both of these genes only appeared in MA. Moreover, 69 genes encoding ribosomal proteins were highly expressed after desalination (Table S2), indicating that protein synthesis is very sensitive to osmolarity changes and that the factors involved in translation could also be affected. As expected, the *nascent polypeptide-associated complex* (*NAC*) was upregulated; NAC can relocalize from a ribosome-associated state to form protein aggregates that act as chaperones to provide the cell with a regulatory feedback mechanism when proteostasis is imbalanced [48]. Moreover, the up-regulated protein kinase C (PRKC) in MA is a Ser/Thr kinase and is known to be involved in gene expression, signal transduction and regulation of the activities of many proteins. This indicates that *E. sinensis* could potentially adjust the concentration of its free amino acid osmolytes for hyper-osmoregulation by amino acid metabolism and protein synthesis as revealed in the adult [13].

#### Stress response

Stress-induced adaptions have been elucidated in several other crustacean species [49] such as *Penaeus monodon* [50] and *Farfantepenaeus paulensis* [51]. In our study, 33 DEGs were detected to be associated with stress adaption (Table 4), with 22 up-regulated genes and 11 down-regulated genes in MA.

#### Salinity stress

In response to salinity stress, the *late embryogenesis abundant-like protein 2* (*LEA2*) disappeared in MA, while *annexin A7* (*ANXA7*) and *corticotropin releasing hormone binding protein* (*CRHBP*) were up-regulated. LEA2 is an extremely hydrophilic protein previously identified in land plants and is involved in their response to desiccation [52]. In the brine shrimp *Artemia franciscana*, another LEA protein is tested to play a role in desiccation tolerance [53]. Therefore, the disappearance of *LEA2* in our study might suggest that with low salinity in the environment, water enter into the bodies of the larvae and thus possibly end water stress. According to our knowledge, *LEA2* is reported in crabs for the first time. ANXA7 can promote membrane fusion and forms calcium channels in membranes [54] by responding to calcium ion and salt stress, while CRHBP is a key regulator of the stress response [55]. Moreover, 11 heat shock protein family members (HSPs) were also down- or up-regulated, including Hsp70, Hsp90, HSC71, HS6B and a dnaJ homolog. As molecular chaperones, their expression has been verified to be influenced by stressful conditions in many other species [12], [56].

Additionally, changes in salinity might kill many larvae due to poor environmental conditions. The stronger larvae might kill the weaker larvae as megalopae, which has been called ‘cannibalism’. Hemolymph coagulation is an essential defense mechanism that can prevent the leakage of hemolymph [57]. In this study, five hemolymph clotting-related protein genes were highly expressed in MA (Table 4), which indicates that after desalination, megalopae might suffer physical injury.

#### Oxidative stress

Oxidative stress is a state of unbalanced tissue oxidation involving enhanced intra- and extra-cellular reactive oxygen species (ROS) production, and often causes a general disturbance of the cellular redox balance. Changes in salinity might cause the production of ROS via an induction of oxidative stress, and the antioxidant enzyme system can scavenge excessive ROS. Under oxidative stress, eight genes were up-regulated in MA, including *glutathione S-transferase* (*GST*), *Ferritins*, *trans-1,2-dihydrobenzene-1,2-diol dehydrogenase* (*DHDH*), *procollagen-lysine,2-oxoglutarate 5-dioxygenase 2* (*PLOD2*) and *metalloprotease* (*MTP*). GST can form a group of multi-gene isoenzymes involved in the cellular detoxification of both xenobiotic and endobiotic compounds, as was reported for other crustacean species [58], [59]. Ferritin is important for iron homeostasis and has ferroxidase activity; it is involved in the conversion of iron from its Fe^2+^ to Fe^3+^ forms, which limits the deleterious Fenton reaction that produces the highly damaging hydroxyl radical [60]. Six genes were down-regulated in MA that principally encoded peroxidase and aldehyde dehydrogenase. Glutathione peroxidase (GPX) is an enzyme family with peroxidase activity whose main biological role is to protect an organism from oxidative damage [61], while aldehyde dehydrogenase plays a crucial role in maintaining low blood levels of acetaldehyde during alcohol oxidation [62]. Among them, the activity of GPX is also found to be influenced by salinity change in the mud crab (*Scylla serrata*) [63]. The different expression levels of these antioxidant enzymes points to a possibility that a variety of oxidative stresses are induced by salinity changes as reported in other decapod crustaceans [64].

### qRT-PCR verification

The DEGs were selected to verify the results of the RNA-Seq analysis by qRT-PCR, using different RNA from those used for RNA-Seq. All the tested genes showed significant differentially expression between MB and MA (Table 1). In the qRT-PCR, *AVP*, *ATP6V1A*, *RAB1A-1*, *SESB* and *PCE* showed high abundance in MA, while *GLYS*, *GLDC* and *LEA2* showed down-regulation in MA. Even though most qRT-PCR results (Table 1) indicated smaller differences (except *GLDC*) compared with the RNA-Seq analysis, there was a consistent expression tendency between the two results, which verified the accuracy of the RNA-Seq.

In conclusion, 908 DEGs were totally detected, especially genes related to ‘Transporters’, ‘Energy and material metabolisms’, ‘Stress adaption’ and the ‘Immune response’. After analysis, we deduced that larvae might compensate for an ion imbalance by adjusting their ion channel and proton pump activities; glyco- and fatty acid metabolism possibly supply energy for the adaption process efficiently; the concentration of free amino acids might be altered via amino acid metabolism and protein synthesis to regulate the concentration of osmolytes. In addition to salinity stress, oxidative stress also occurred, to which the megalopae of *E. sinensis* showed a complete set of adaption mechanisms. Therefore, the megalopae of *E sinensis* showed a similar hyper-osmoregulation mechanism as the adult. However, no significant expression differences were observed in *Na^+^/K^+^-ATPase*; *LEA2*, a hydrophilic protein, was found disappeared after desalination, which was reported in crabs for the first time. This study is expect to provides useful genetic resources for further research on crab larvae osmoregulation and their stress-induced responses to environmental changes.

## Supporting Information

Figure S1 **Length distribution of the unigenes in the transcriptomes of** ***Eriocheir sinensis*** **.**
(TIF)

Figure S2 **GO distribution of all of the unigenes in the transcriptomes of** ***Eriocheir sinensis*** **.**
(TIF)

Figure S3 **eggNOG functional distribution of all of the unigenes in the transcriptomes of** ***Eriocheir sinensis*** **.**
(TIF)

Figure S4 **Functional distribution of differentially expressed genes in the MB and MA transcriptomes of** ***Eriocheir sinensis*** **based on KEGG analysis.**
(TIF)

Figure S5 **Differentially expressed functional processes based on the KEGG analysis. The horizontal line indicates the significance threshold (** ***p*** **<0.05).**
(TIF)

Figure S6 **eggNOG functional distribution of differentially expressed genes in the MB and MA transcriptomes of** ***Eriocheir sinensis*** **.**
(TIF)

Table S1 **Summary of the transcriptomes from the megalopae of** ***Eriocheir sinensis*** **before (MB) and after (MA) desalination.**
(DOCX)

Table S2 **Differentially expressed genes from the transcriptomes of MB and MA in** ***Eriocheir sinensis*** **.**
(XLSX)

Checklist S1 **The ARRIVE (Animal Research: Reporting of** ***In Vivo*** **Experiments) Guidelines Checklist.**
(DOC)
