# Supplementary figures and images for: Transcriptome Changes in Eriocheir sinensis Megalopae after Desalination Provide Insights into Osmoregulation and Stress Adaption in Larvae
Source: PLoS One. 2014 Dec 3;9(12):e114187. doi: 10.1371/journal.pone.0114187 (PMC4254945; doi:10.1371/journal.pone.0114187)

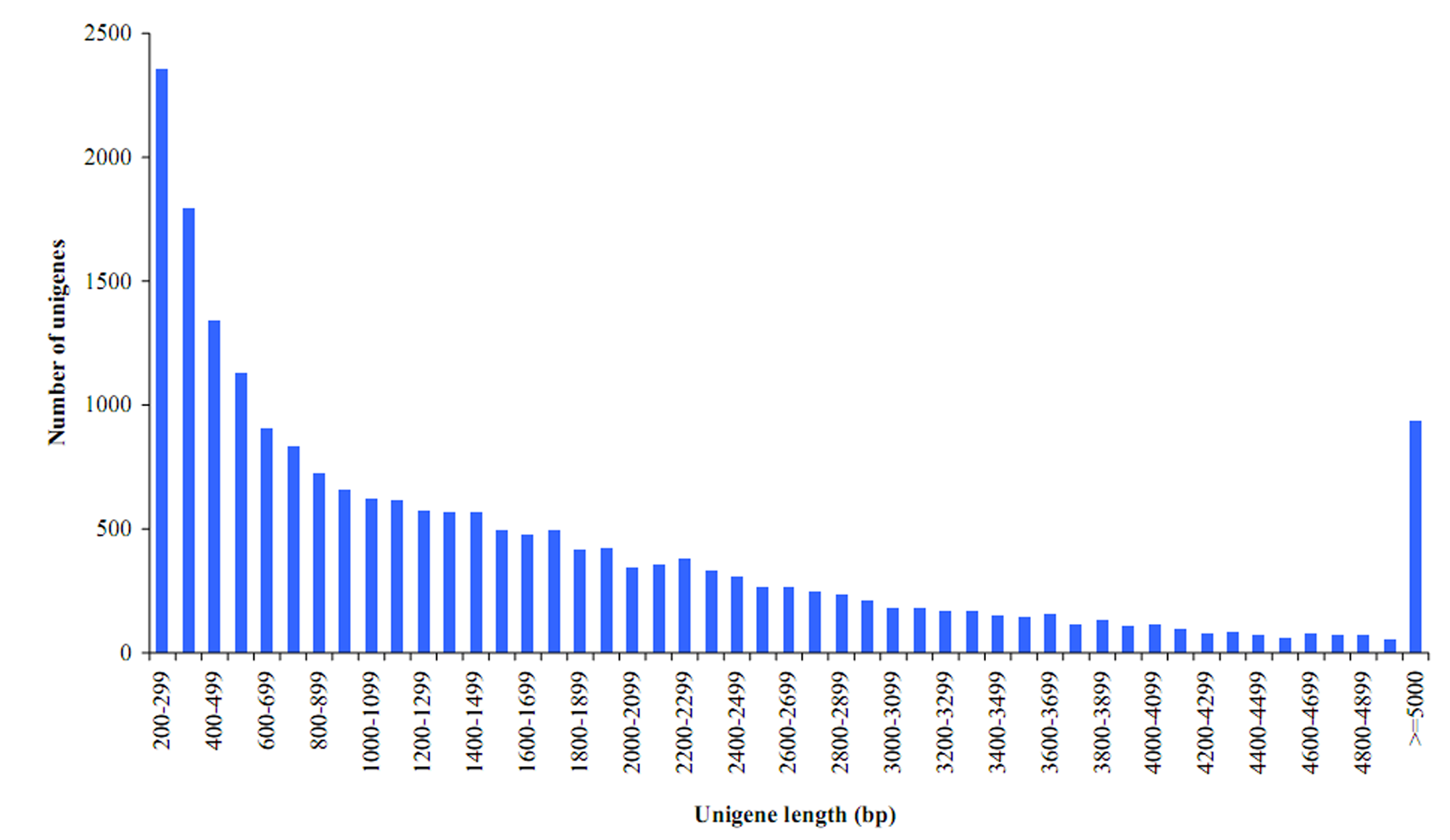

Supplement: Figure S1 — Length distribution of the unigenes in the transcriptomes of Eriocheir sinensis . (TIF) [file pone.0114187.s001.tif]

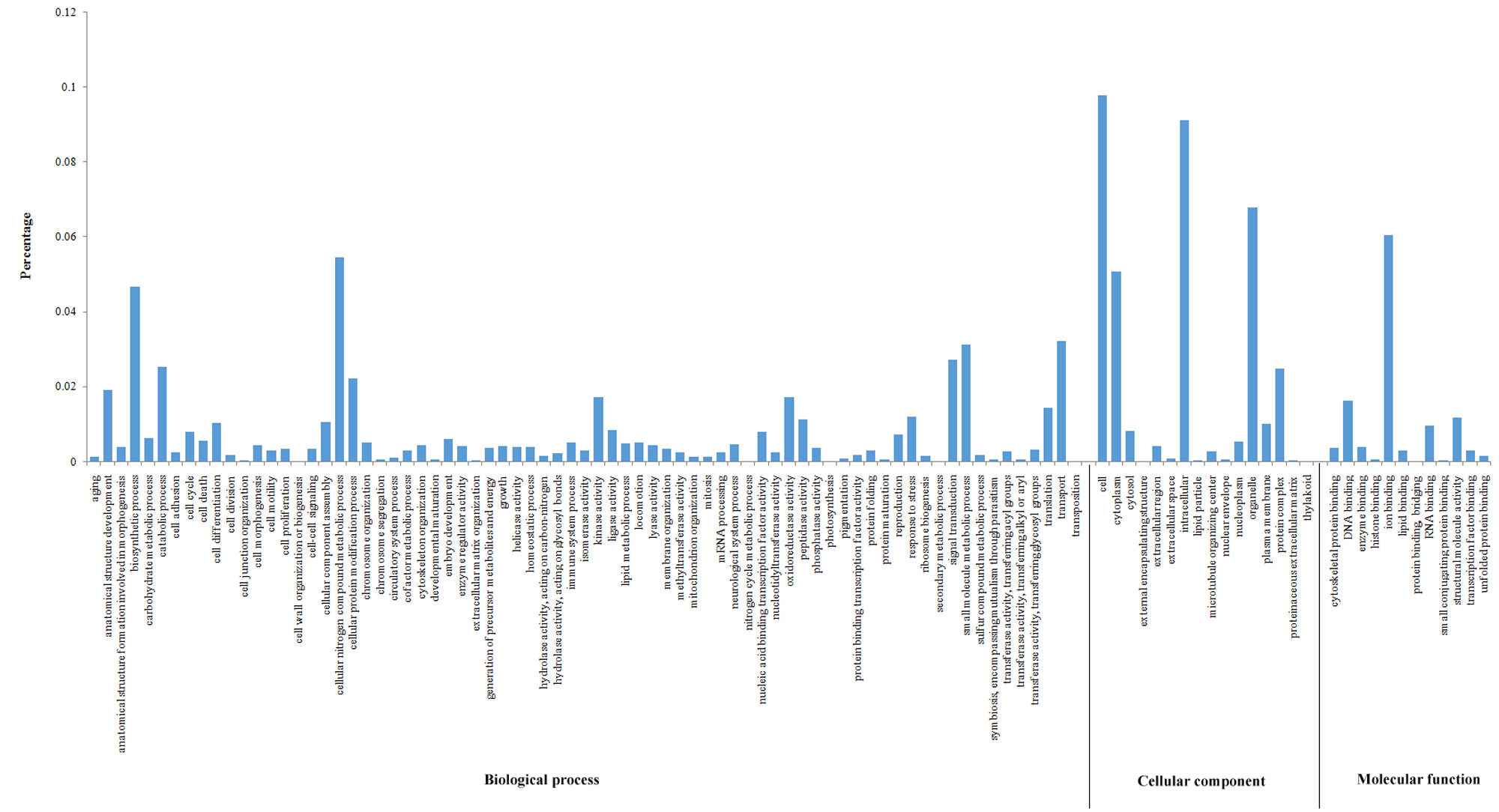

Supplement: Figure S2 — GO distribution of all of the unigenes in the transcriptomes of Eriocheir sinensis . (TIF) [file pone.0114187.s002.tif]

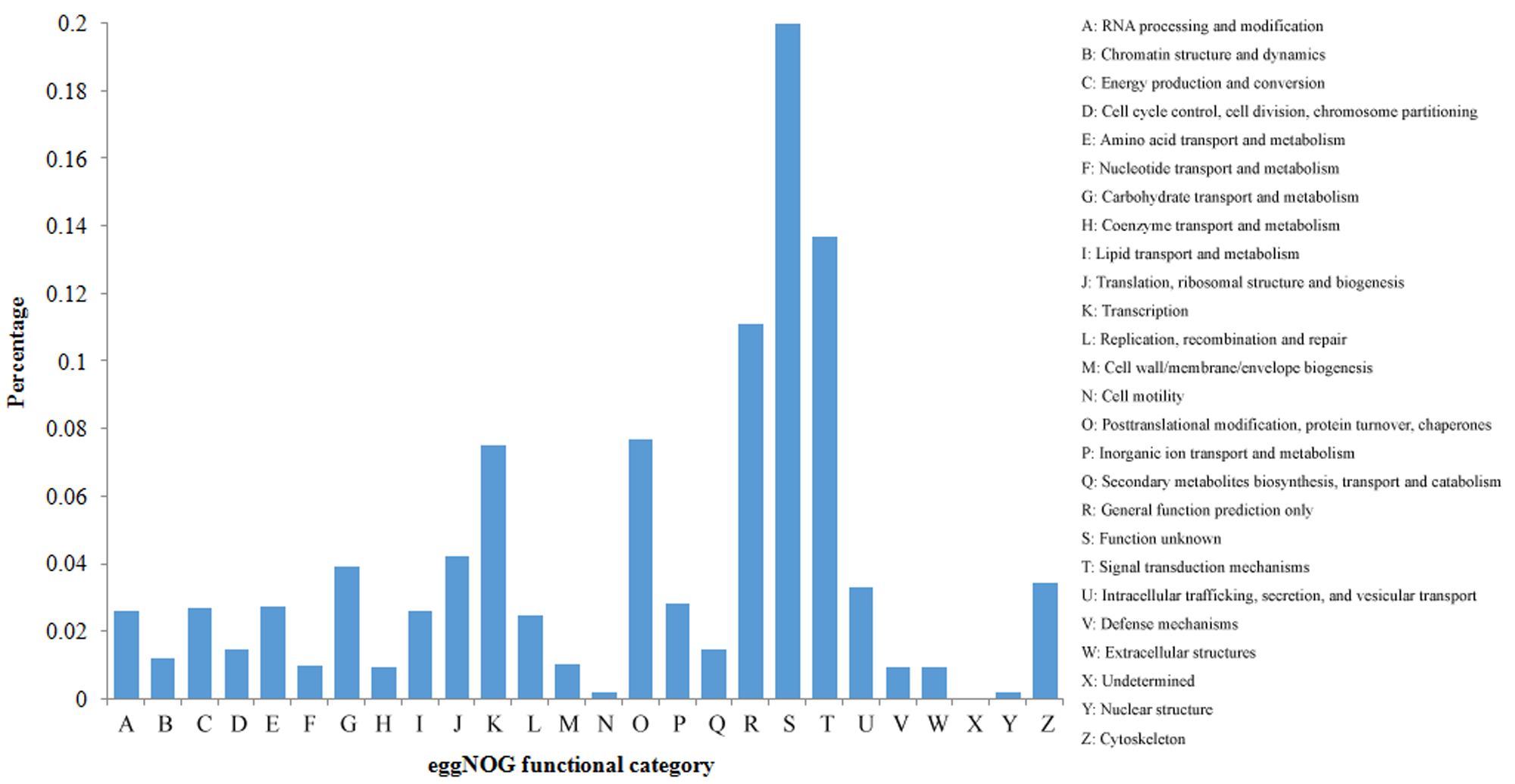

Supplement: Figure S3 — eggNOG functional distribution of all of the unigenes in the transcriptomes of Eriocheir sinensis . (TIF) [file pone.0114187.s003.tif]

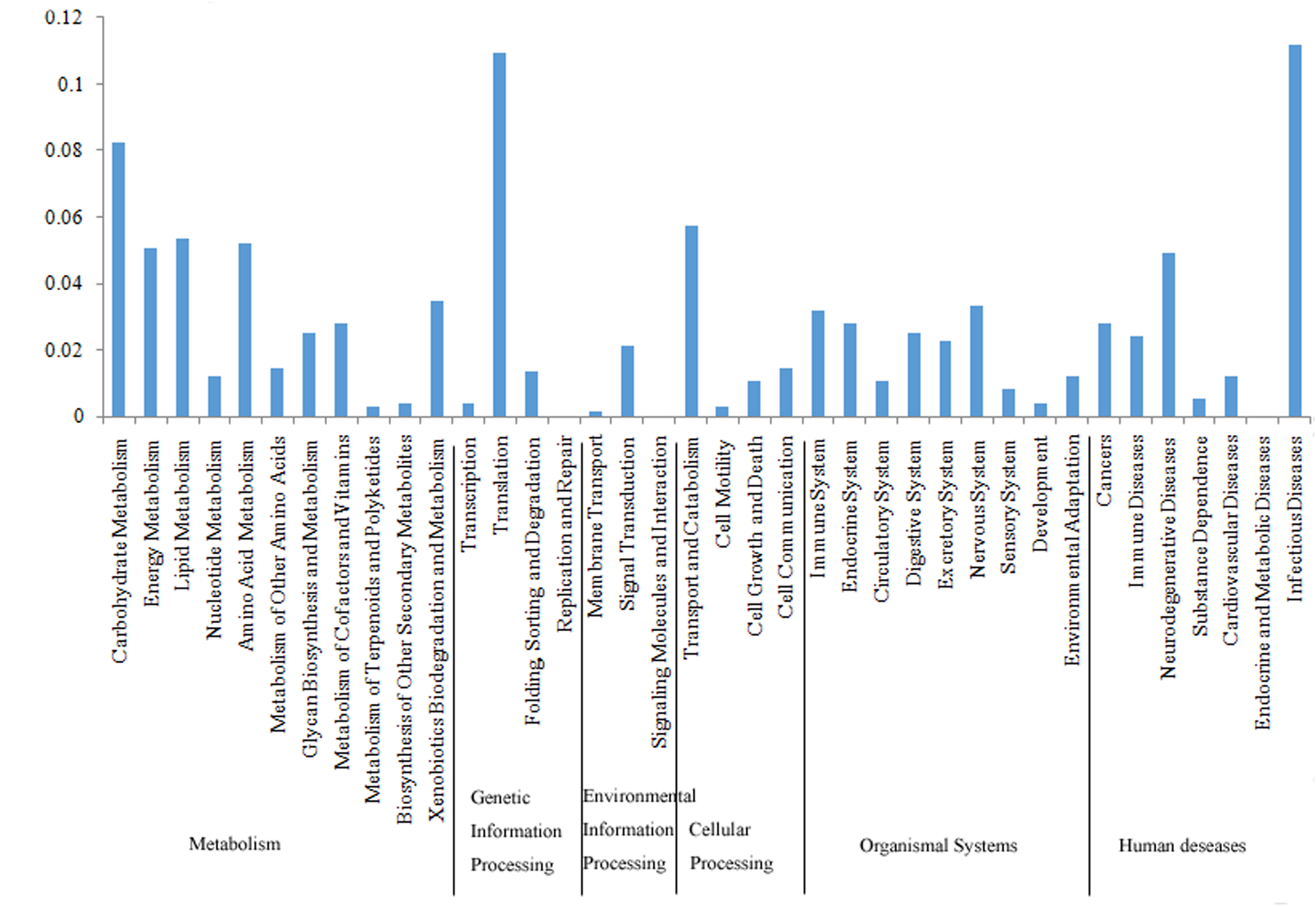

Supplement: Figure S4 — Functional distribution of differentially expressed genes in the MB and MA transcriptomes of Eriocheir sinensis based on KEGG analysis. (TIF) [file pone.0114187.s004.tif]

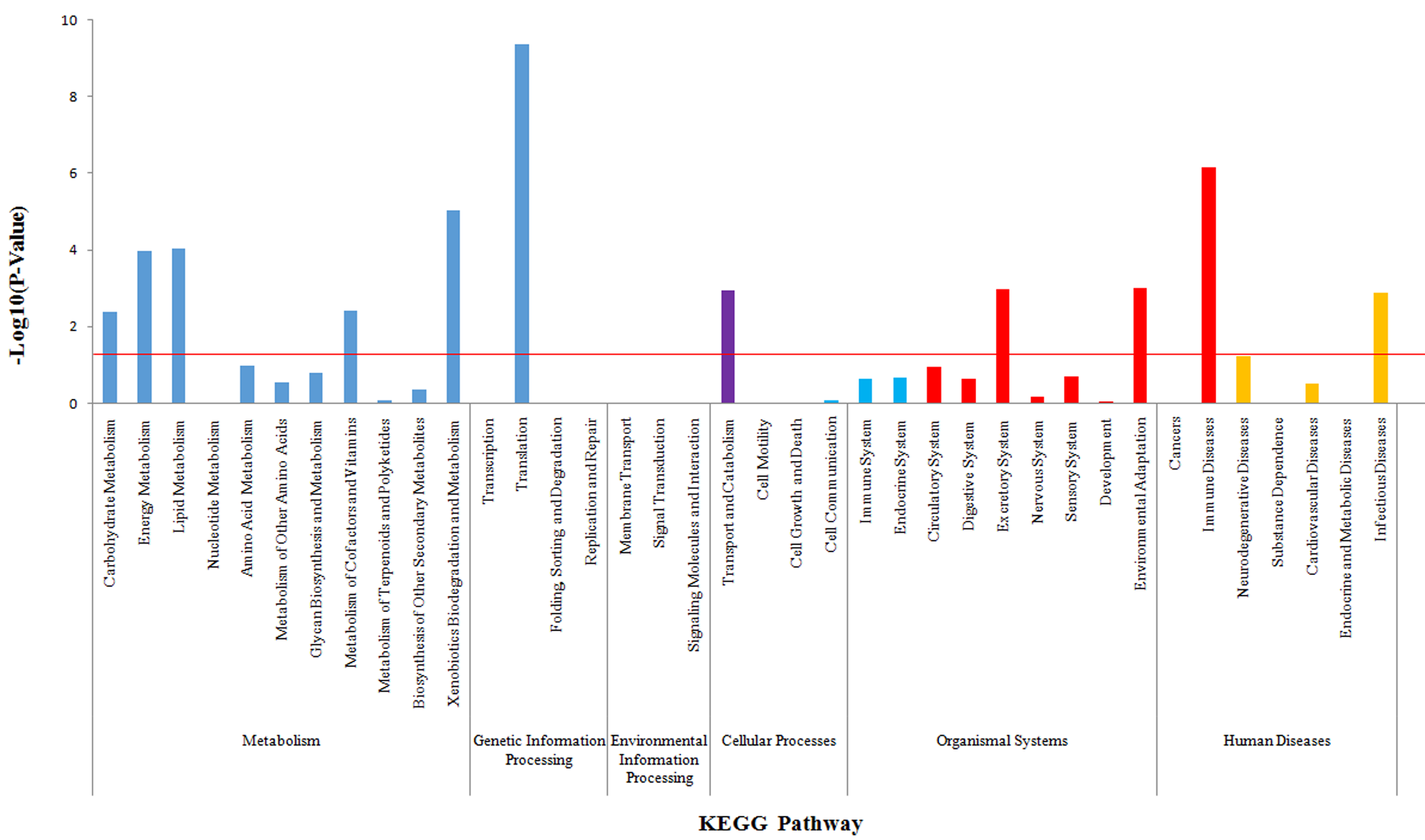

Supplement: Figure S5 — Differentially expressed functional processes based on the KEGG analysis. The horizontal line indicates the significance threshold ( p <0.05). (TIF) [file pone.0114187.s005.tif]

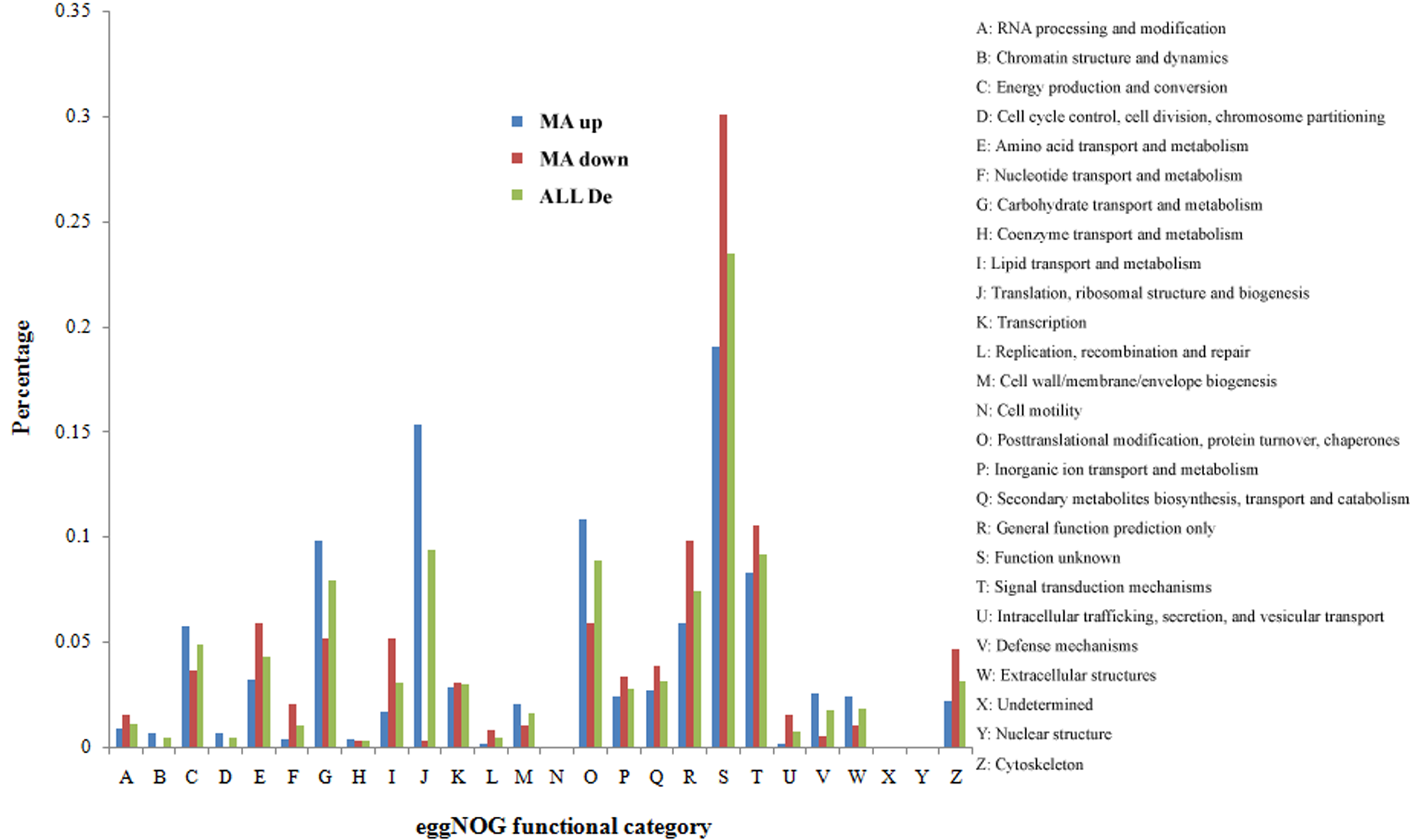

Supplement: Figure S6 — eggNOG functional distribution of differentially expressed genes in the MB and MA transcriptomes of Eriocheir sinensis . (TIF) [file pone.0114187.s006.tif]
