# Supplementary material for: Transcriptome Changes in Eriocheir sinensis Megalopae after Desalination Provide Insights into Osmoregulation and Stress Adaption in Larvae
Source: PLoS One. 2014 Dec 3;9(12):e114187. doi: 10.1371/journal.pone.0114187 (PMC4254945; doi:10.1371/journal.pone.0114187)
Supplement: Table S1 — Summary of the transcriptomes from the megalopae of Eriocheir sinensis before (MB) and after (MA) desalination. (DOCX) [file pone.0114187.s007.docx]

**Table S1.** Summary of the transcriptomes from the megalopae of *Eriocheir sinensis* before (MB) and after (MA) desalination.

| **Separate transcriptome data** | MB | MA |
| --- | --- | --- |
| Raw reads | 63,433,542 | 61,764,914 |
| Q20 percentage % | 97.35 | 97.41 |
| GC percentage % | 51.64 | 49.69 |
| Clean reads | 58,339,580 | 56,923,344 |
| Total clean base pairs (Gb) | 5.70 | 5.56 |
| **Assembling of the two transcriptome** | MB & MA | |
| Total number of contigs | 243,345 | |
| Mean length of contigs (bp) | 367 | |
| N50 of contigs (bp) | 648 | |
| Total number of transcripts | 127,983 | |
| Mean length of transcripts (bp) | 1,018 | |
| N50 of transcripts (bp) | 2,314 | |
| **Annotation with different databases** | MB & MA | |
| Total number of annotated unigenes | 21,042 | |
| Mean unigene length (bp) | 1,635 | |
| N50 of Unigenes (bp) | 2,629 | |
| Annotation with NR | 21,042 | |
| Annotation with GO | 7,499 | |
| Annotation with eggNOG | 20,139 | |
| Annotation with KO | 8,653 | |
| Annotation with EC | 3,508 | |

NR: non-redundant protein sequences in NCBI; GO: Gene Onotology; eggNOG: evolutionary genealogy of genes: Non-supervised Orthologous Groups; KO: KEGG (Kyoto Encyclopedia of Genes and Genomes) Orthology; EC: Enzyme Commission number.
